# Detection of fake news and hate speech for Ethiopian languages: a systematic review of the approaches

**DOI:** 10.1186/s40537-022-00619-x

**Published:** 2022-05-19

**Authors:** Wubetu Barud Demilie, Ayodeji Olalekan Salau

**Affiliations:** 1 Department of Information Technology, Wachemo University, Hossana, Ethiopia; 2 Department of Electrical/Electronics and Computer Engineering, Afe Babalola University, Ado-Ekiti, Nigeria

**Keywords:** Artificial intelligence, Ethiopian languages, Deep learning, Fake news, Hate speech, Machine learning, Social media platform

## Abstract

With the proliferation of social media platforms that provide anonymity, easy access, online community development, and online debate, detecting and tracking hate speech has become a major concern for society, individuals, policymakers, and researchers. Combating hate speech and fake news are the most pressing societal issues. It is difficult to expose false claims before they cause significant harm. Automatic fact or claim verification has recently piqued the interest of various research communities. Despite efforts to use automatic approaches for detection and monitoring, their results are still unsatisfactory, and that requires more research work in the area. Fake news and hate speech messages are any messages on social media platforms that spread negativity in society about sex, caste, religion, politics, race, disability, sexual orientation, and so on. Thus, the type of massage is extremely difficult to detect and combat. This work aims to analyze the optimal approaches for this kind of problem, as well as the relationship between the approaches, dataset type, size, and accuracy. Finally, based on the analysis results of the implemented approaches, deep learning (DL) approaches have been recommended for other Ethiopian languages to increase the performance of all evaluation metrics from different social media platforms. Additionally, as the review results indicate, the combination of DL and machine learning (ML) approaches with a balanced dataset can improve the detection and combating performance of the system.

## Introduction

In recent years, artificial intelligence (AI) has brought about significant changes in the domain of information technology and other disciplines, such as the use and development of intelligent transportation systems, virtual personal assistants, robotic surgery, and most significantly, natural language processing (NLP) applications [1]. Accordingly, the world is rapidly changing in technological aspects. The digital world provides several benefits and drawbacks. One of its drawbacks is fake news and hate speech, which is incredibly simple to spread. Fake news and hate speeches are defined as intentionally and verifiably false news [2–4]. Individuals, governments, freedom of speech, news systems, and society are all becoming increasingly vulnerable to it. The rising use of social media and knowledge sharing has benefited humanity considerably. Today, social media platforms have a significant impact on people’s daily lives [5]. Such social media platforms like Facebook and Twitter have aided in the spread of rumors, conspiracy theories, hatred, xenophobia, racism, and prejudice [6].

While technology has many advantages, it can also influence public opinion and religious views all over the world. It can be used both directly and indirectly to target people based on race, caste, ethnic origin, religion, ethnicity, nationality, sex, gender identity, sexual orientation, handicap, or sickness.

The social media sphere, which has long been tightly controlled by the Ethiopian government, appears to be untying itself following the start of the new political reforms in 2018 [7]. Following the transition of the government, it is clear that people are enjoying greater freedom of expression. On the contrary, the emergence of hate speech attributed to political, ethnic, and religious underpinnings is said to have subdued the new digital platform.

Ethiopia’s social media landscape was changing faster than you could refresh your page in 2021 [8]. It was rife with controversies, disinformation, and organized social media campaigns, owing primarily to major political events that occurred during that period.

The World Health Organization (WHO) warned against fake news and hate speech in the COVID-19 infodemic [9, 10] to indicate the proliferation and negative impact of fake news and hate speech during the current pandemic and that they are a huge threat to democracy and political stability. Along with the COVID-19 pandemic has emerged an infodemic of false and misleading information, complicating COVID-19 response efforts. It was stated that as the virus spreads, misinformation makes the job of the brave health workers even more difficult; it diverts the attention of the decision-makers, causes confusion, and spreads fear among the general public; and the list of practical examples of the effects of fake news and hate speech is growing, and the danger is already imminent [11, 12].

Governments, the technology industry, and individual researchers have all tried to come up with ways to mitigate the negative impacts of fake news and hate speech. As a result, some governments have attempted to pass legislative declarations that they hope will suppress fake news and hate speech. For example, Ethiopia’s government has enacted the hate speech and disinformation prevention and suppression proclamation No. 1185/2020 [13]. Ethiopia’s cabinet has approved a notice to combat fake news and hate speech, which includes expanding Facebook’s third-party fact-checking to Ethiopia and other African countries [14, 15]. According to the proclamation of [16], article 19, is concerned about the wording and application of Ethiopia’s hate speech and disinformation laws against those who oppose the government’s policies. The proclamation to prevent the spread of hate speech and false information, which went into effect on March 23, 2020, is extremely problematic from the standpoint of human rights and free speech and should be immediately revised. In any case, while the proclamation is still in effect, it must not be abused, and the government must not abuse its power under the guise of dealing with the public health crisis. Ethiopians now have unprecedented civil and political liberties because of the country’s new government. When the press and broadcast media were censored in previous years, social media gave Ethiopians, like many others around the world, the freedom to speak, organize, mobilize, and challenge the government’s narrative. Despite these changes, one thing has remained constant: authorities continue to challenge the relative “freedom” that social media platforms have enabled. While the previous administration surveilled, blocked, and punished dissenting voices online, prime minister Abiy’s administration has enacted the hate speech and disinformation prevention and suppression proclamation, which gives the government the authority to fine and imprison citizens for their social media activities [17].

To address hate speech and disinformation, which have historically troubled the country, Ethiopia enacted the hate speech and disinformation prevention and suppression proclamation in March 2020 [18]. However, while government regulation is necessary to control hate speech, Ethiopia’s new law threatens online freedom of expression and access to information.

As a result, it seems to be less useful, as fake news and hate speech creators conceal their work, leaving no record for the law. Using various methods, Facebook, Google, Twitter, and YouTube tried to take technological precautions.

Linguistic resources are vital in the creation of fake news and hate speech detection approaches. However, “low-resource” languages, primarily African languages, lack such tools and resources [11]. Ethiopia has established a policy to introduce four more working languages in addition to Amharic, which has traditionally served as the country’s working language. The government will adopt Afan Oromo, Ethiopia’s most frequently spoken language, as well as Afar, Somali, and Tigrigna as official languages in the future [19]. Despite this, Ethiopian languages remain among the world’s “low-resource” languages, lacking the tools and resources required for natural language processing applications and other techno-linguistic activities. However, a lack of appropriate datasets and good word embedding have made it difficult to create detection techniques that are reliable enough [11]. Recent improvements in natural language processing and understanding have made it possible to detect and counteract fake news and hate speech in textual streams with greater accuracy by using different approaches.

With the growing influence of social media platforms in affecting public opinion and ideas around the world, there has been a greater focus on recognizing and combatting fake news and hate speech on various platforms [20]. Currently, in Ethiopia, hate speech and the spread of fake news have already impacted the lives of millions of people. Some schools, public and private universities, or colleges have recently closed; business activities have been severely hampered due to the closure of major roads in the country; citizen movement has been severely hampered; millions have been displaced, and many thousands have died due to scarcity of food and shelter [21].

Accordingly, all Ethiopians are suffering more from the harmful effects of social media, than those in other developing countries [21]. As described in [22], fighting against fake news and hate information is to save lives. Fake news, misinformation, and hate speech have flourished in Ethiopia’s media ecosystem, especially in online systems [23]. This is strongly linked to significant, tragic, real-world consequences, which exacerbated pre-existing tensions and contributed to violence and conflict. To date, the Ethiopian government’s response to the spread of fake news, misinformation, and hate speech has been heavy-handed, with the go-to response to escalation being to turn off the internet for the entire country. However, as the Internet and social media communications, such as Twitter, YouTube, and Facebook messages, have evolved, so have the chances and obstacles to developing such solutions. The fake news and hate speech detection method used to detect and counteract fake news and hate speech on social media is far from flawless [24].

For foreign and Ethiopian languages, several studies have been undertaken to detect and counteract fake news and hate speech on various social media platforms. Researchers have been conducted to detect and combat fake news and hate speech from various social media for Ethiopian languages [11, 19, 20, 25, 26] and have advised future researchers to collect more corpora from various sources and use different approaches to improve the performances of the system in detecting and combatting of fake news and hate speech from various social media platforms. This study is planned to review the implemented approaches for fake news and hate speech detection research works in Ethiopian languages and to recommend the best approach regarding the performances of the evaluation metrics for future researchers of the area to minimize the risks that come due to the widespread of fake news and hate speech among the societies.

The rest of the paper is organized into different but interrelated sub-sections. The paper begins by discussing the related works in "Related works" section, results and discussions in "Results and discussion" section, and the paper is a conclusion and future works in "Conclusion and recommendation" section.

### Related works

In recent years, there has been an increase in scientific interest in detecting and combating fake news and hate speech. This was caused by the spread of hatred and other negative emotions on social media platforms. The Amharic language fake news classification and detection on social media have been developed by using the ML approach [5]. The author has proposed an AI method to develop a solution to fake news on the internet. The review attempted to explicitly create, execute, and consider AI and text highlight extraction techniques for counterfeit news recognition in the Amharic language. The discussion expanded on the current online media administrations for detecting fake news.

Authors [11] investigated the identification of fake news in the Amharic language using DL approaches, and news content, as well as developing many computational linguistic tools for these “low-resource” African languages.

DL approaches and word embedding were employed by the researchers to develop automatic fake news detection mechanisms. A general-purpose Amharic corpus (GPAC), a novel Amharic fake news detection dataset (ETH FAKE), and Amharic fasttext word embedding are among the contributions. As a result, the Amharic fake news detection model was evaluated using the ETH FAKE dataset and performed exceptionally well when utilizing the Amharic fasttext word embedding (AMFTWE). Using both word embeddings, cc-am-300 and AMFTWE, the fake news detection model performed exceptionally well. When using the 300 and 200 dimension embeddings, the model had a validation accuracy of above 99%. They have included the experimental results of the model performance utilizing the cc-am-300 and AMFTWE embeddings, which were with an accuracy of 99.36%, precision of 99.30%, recall of 99.41%, and an f1-score of 99.35%. Finally, they suggested using other word embedding approaches, such as bidirectional encoder representations from Transformers (BERT), which could help train a word embedding possibly better than AMFTWE if BERT’s data-hungry nature was satisfied, even if creating an Amharic fake news dataset and obtaining many Amharic corpora would be difficult.

The work [27] has outlined that developing hate speech detection for Afan Oromo social media is essential to eliminate the risk of hate speech on social welfare. They have conducted experiments six times by applying ML approaches such as support vector machine (SVM), multinomial Naïve Bayes (MNB), linear support vector machine (LSVM), logistic regression (LR), and random forest (RF) classifier to build hate speech detection prototypes for Facebook and Twitter platforms.

Even though, they have developed the Afan Oromo hate speech detection model using ML approaches by collecting data from Facebook and Twitter platforms. The study only investigated posts and comments in textual documents. The posts and comments in the form of images or photos, audio, or video data have not been considered. Researchers employed performance criteria like accuracy, precision, recall, and f1-score to evaluate the performance. ML feature selection approaches such as bigram and term frequency-inverse document frequency (TF-IDF) were used. According to the findings, SVM achieved an LSVM performance precision of 66%, a recall of 66%, and an f1-score of 64%. The precision of 60%, recall of 65%, and f1-score of 62% were all reached with the MNB. A precision of 64%, a recall of 64%, and an f1-score of 63% were achieved for the RF classifier. Performance precision was 65%, a recall was 64%, and f1-score was 61% for the LR classifier. Accordingly, the SVM achieved a performance precision of 66%, a recall of 65%, and an f1-score of 63%. They analyzed its performance and discovered that LSVM has the highest precision, recall, and f1-score values of 66%, 66%, and 64%, respectively. Therefore, the researchers agreed to use LSVM to deploy the Afan Oromo hate speech detection model.

The most important limitation of this study lies in applying conventional ML approaches that need manual labeling of the dataset. The experiments conducted on the data were small. They have recommended and concluded the research work as future research can also be conducted by collecting data from other social media platforms. In addition to collecting data from other social media platforms, researchers can consider other modes of data collection for further research to be investigated.

According to those researchers, going beyond conventional ML approaches for experiments can also be the next study. Another research work [25] came up with the Afan Oromo fake news detection system. The proposed system includes preprocessing such as tokenization, normalization, stop word removal, and abbreviation resolution, feature extraction such as term frequency-inverted document frequency (TF-IDF), term frequency (TF), and hash to determine word importance in the news and the corpus, and N-grams, a powerful natural language processing technique for capturing semantic and syntactic information. With a passive-aggressive classification system, all conceivable combinations of feature extraction techniques and natural language processing approaches were applied. According to the study, passive-aggressive (PA) outperforms ensemble methods like gradient boosting and random forest, as well as linear classifiers like MNB. The PA outperforms with 97.2% and an error of 2.8%. Finally, utilizing the TF-IDF feature extraction using Unigram and PA classification approaches, a Python Django was utilized for the web-based deployment of the model. Despite the dataset’s shortcomings, the linear PA with TF-IDF vector and unigram model outperforms the competition with 97.2% of precision, 97.9% of recall, and 97.5% receiver operating characteristic area under the ROC Curve (ROC AUC) f1-score.

Using a DL system, the work [28] aimed to detect Amharic language fake news. They employed a newly acquired dataset to complete their research because there were no previously available resources in the area they wanted to investigate. They used the graph application programming interface (API) to collect data from the Facebook platform, and two journalists annotated the dataset. To ensure that the data is uniformly annotated across various annotators, guidelines from the news literacy project were used, resulting in an annotated dataset of 12,000 stories with a binary class. They have used equal-sized class instances, 6,000 for each fake and genuine class, to avoid an issue with an imbalance in the number of instances in each class and to be dependable on classification reports. With an accuracy of 93.92%, a precision of 93%, recall of 95% (which is smaller than bidirectional long short-term memory’s (Bi-LSTM’s) 96%), and an f1-score of 94%, the convolutional neural network (CNN) model outperform all other models. The impact of morphological normalization on Amharic fake news identification was investigated using the top two performing models, and the results demonstrated that normalization harms classification performance, lowering both models’ f1-score from 94 to 92%. Finally, CNN was shown to be the most effective model in the investigation.

Furthermore, contrary to their expectations, the attention mechanism used in the sequential models performs worse than the baseline model. Another finding of the study was that in the Amharic language fake news dataset, morphological normalization was not always helpful in improving model performance. According to this study, evaluating different approaches from other disciplines, such as capsule networks (CN), would be a good idea. The CN is doing better in the world of computer vision, and applying their strength to the NLPA challenges could assist in improving the Amharic language fake news and hate speech detection model. Furthermore, they recommend that researchers interested in this field should have to train their embeddings with domain-specific data to obtain a more semantically strong embedding model, which could lead to better detection. According to [29], DL approaches have recently gained a lot of attention and have improved the state-of-the-art for many difficulties that artificial intelligence and ML approaches have faced for a long time. The goal of the research was to provide a method for detecting fake news on social media using the DL approach for Afan Oromo news text. A model to predict and classify Afan Oromo news text must be preprocessed and trained on the sample dataset. As a result, the researchers looked at one hot encoder for mapping category integers and used it in the context of word embedding by training it with Bi-LSTM and a cosine similarity measure, which are supplied as input features to the neural network (NN). After the classifier was trained to classify, a 0.5 threshold was applied to the output score to decide whether it was true or fake, and statistical analysis, a confusion matrix was used to compare across different thresholds, and the suggested model necessitated a large amount of data.

However, when compared to the dataset created for the English language, the dataset in the Afan Oromo language is a major concern; the model is trained on very minimal data. Boosting the consistency of the performance by adding data to the news dataset would increase user trust in the system. On a benchmark dataset, the model can predict with an accuracy of 90%, precision of 90%, recall of 89%, and an f1-score of 89%, outperforming the current state of the art applying the Bi-LSTM model. Finally, they concluded that the Bi-LSTM system prototype can be used as a foundation for future work with the Afan Oromo news text datasets and other Ethiopian local languages. Another work on Afan Oromo text content-based fake news detection using MNB [19] found that the best performing models were an MNB Classifier with word frequency, feature extraction, and unigram, which had a classification accuracy of 96%. The model was tested using 0.7 thresholds, which may not be the most reliable for models with poorly calibrated probability scores. term frequency performs better, yet frequent but not crucial terms have an impact on the outcome. These obstacles limited the scope of the study and prevented it from being more broadly applicable. They used TF, TF-IDF, and TF-IDF) of unigram and bi-grams, and discovered that the term frequency of unigram of this model identifies fake news sources with 96% accuracy, with only minor effects on recall. For real news accuracy, recall, and f1-score, the confusion matrix was computed at 98.6%, 94%, and 96.2%, respectively, and for fake news precision, recall, and f1-score, at 91%, 97.8%, and 94%, respectively. As a result, it was decided that these difficulties, as well as slang phrases, would be addressed in future work. According to [26], social media platforms’ quick growth and expansion have filled the information-sharing gap in everyday life. The Amharic language fake news dataset was created using verified news sources and social media pages, and six different ML approaches were designed, including Naïve Bayes (NB), SVM, LR, SGD, RF, and PA Classifier. The experimental results show a precision of 100% RF for both TF-IDF and Count Vectorizer (CV), a recall of 95% using the PA classifier for TF-IDF, and an f1-score of 100% in NB and LR classifier for TF-IDF vectorizer using PA classifier. The research has made a substantial contribution to slowing the spread of misinformation in vernacular languages. The work [30] sought to create, implement, and analyze hate speech detection systems for the Amharic language using ML approaches and text feature extraction. According to the study, it was critical to comprehend and define hate and offensive speech on social media, investigate existing techniques for addressing the issues and comprehend the Amharic language in-depth, as well as the various methods used to implement and design models capable of detecting hate speech. Collecting posts and comments for the dataset, defining annotation rules, preprocessing, features extraction using N-gram, TF-IDF, and word2vec, model training using SVM, NB, and RF, and model testing are some of the approaches used. The experiment produced twenty-one (21) binary and ternary models for each dataset utilizing two datasets. Both SVM and NB were outperformed by binary models that used RF with word2vec. The SVM with word2vec, on the other hand, outperforms NB and RF models in classification with a 73% f1-score. In addition, the ternary SVM model using word2vec produced a 53% f1-score, which is better than the NB and RF models. Finally, in both datasets utilized in this study, models based on SVM employing word2vec performed marginally better than NB and RF models. The work [31] uses LSTM and GRU with word N-grams for feature extraction and word2vec to represent each unique word by vector representation to construct recurrent neural network (RNN) models for automated hate speech post identification from the Amharic language posts and comments on Facebook. To train the model and identify the optimum hyper-parameters combination for automated hate speech post and comment detection, an experiment was done on the two models, utilizing 80% of the data set for training and 10% for validation. The remaining 10% of the dataset was utilized to test the model after it had been trained. As a result, by training 100 epochs, an LSTM-based RNN with batch size 128, learning rate of 0.1%, RMSProp optimizer, and 0.5 dropouts achieves an accuracy of 97.9% in detecting posts as hate speech or free. This was ensured by applying the model’s performance test and inference on user-generated data to test the models. The RNN-LSTM model produced an improved test accuracy of 97.9% when used with this dataset and different parameters on GRU and LSTM based RNN models by feature representation of word2vec. Finally, they found that using DL neural network models for the Amharic language text data analysis allowed them to detect hate speech posts on the Facebook platform, with LSTM outperforming GRU on their dataset. The accuracy of the DL approach is affected by changes in neural network hyperparameters. The research work [21] reported on an examination of the first Ethiopic Twitter dataset for the Amharic language, which was aimed at detecting abusive speech. The researchers evaluated the distribution and trend of abusive speech material over time, compared abusive speech content from Twitter and a general reference Amharic language corpus, and gathered 144 abusive speech keywords from five native speakers of the language and classified them as hate and offensive speech.

The research work [32] created an apache spark (AS) model to categorize the Amharic language Facebook posts and comments into hate and non-hate categories. For learning, the authors used RF and NB, and for feature selection, they used Word2Vec and TF-IDF. The NB classifier with the word2Vec feature model outperformed the Facebook social network for Amharic language posts and comments regarding the accuracy, ROC score, and the area under precision and recall, with 79.83%, 83.05%, and 85.34% of accuracy, ROC score, and area under precision and recall, respectively. For the TF-IDF feature model, the NB achieves better results with 73.02%, 80.53%, and 79.93% for accuracy, ROC score, and area under precision and recall, respectively. The RF with word2vec feature outperforms the TF-IDF with accuracy, ROC score, and area under precision and recall of 65.34%, 70.97%, and 73.07% respectively. The TF-IDF is next with 63.55%, 68.44%, and 69.96% of accuracies, respectively. In [33], a model for detecting hate speech and identifying vulnerable communities in Amharic texts on the Facebook platform was developed. They gathered the Amharic language postings and comments from questionable public profiles of organizations and individuals on social media. To get a clean corpus, the necessary preprocessing was done according to the language’s requirements. The word embedding (Word2Vec) model was then trained, and human annotators were chosen to label texts using the standards and norms that have been provided. Following that, in the AS environment, feature extraction approaches using Word2Vec word embedding controlled by TF-IDF, TF-IDF alone, and word N-grams were used. In their trials, the RNN-LSTM and RNN-GRU DL approaches were compared to the standard GBT and RF approaches. The best performances were achieved using Word2Vec embedding and RNN-GRU, which had an AUC of 97.85% and an accuracy of 92.56% in the hate speech detection experiments. Finally, they suggest that other inherent problems in the RNN can be solved with a more powerful architecture (that can handle negation and use information throughout the posts and comments), such as tree-LSTM, which can learn meanings from characters and parts of words, rather than word tokens themselves, as they have done. Automatic hate and offensive speech detection framework from social media have been implemented for the Afan Oromo language [34]. The overall goal of this study was to create a framework for categorizing hate and neutral speech. The researchers recommended using SVM with TF-IDF, N-gram, and W2vec feature extraction to create a binary classifier dataset for detecting hate speech in the Afan Oromo language. To create the dataset for this study, they used Face Pager and Scrap Storm API to scrape data from Facebook posts and comments. Following data gathering, they divided the information into two categories: hatred and neutrality. Additionally, when they compared the outcomes of several ML approaches, accuracy, f-score, recall, and precision measurements were used to evaluate the experiment.

In all evaluation measures, the framework based on SVM with N-gram combination and TF-IDF achieves a performance of 96% (accuracy, f1-score, precision, and recall). A summary of the related work that has been used in this review work is presented in Table 1.

**Table 1:** Summary of related works for Ethiopian languages

Author (s)	Approaches	Contributions of Author (s)	Evaluation Metrics
[11]	DL and word embedding	✓ They have collected and arranged a sizable Amharic corpus for general use. ✓ They’ve developed an Amharic fasttext word embedding system. ✓ They’ve created a brand-new dataset for detecting bogus news in Amharic. ✓ They’ve developed a DL strategy for detecting bogus news in Amharic. ✓ They ran a series of tests to see how well the word embedding and fake news detection models worked.	✓ Using both word embedding, cc-am-300 and AMFTWE, the fake news detection model performed exceptionally well. ✓ When using the 300 and 200-dimension embedding, the model had a validation accuracy of above 99%. ✓ Finally, they included the experimental results of the model performance utilizing the cc-am-300 and AMFTWE embedding, which were accuracy of 99.36%, precision of 99.30%, recall 99.41%, and f1-score of 99.35%.
[27]	ML (i.e., SVM, MNB, LSVM, LR, DT, and RF)	✓ They built a model that detects Afan Oromo hate speech on social media using a combination of n-gram and TF-IDF feature extraction methodologies. ✓ They collected 13,600 comments and posts on respective public pages using Facepager (https://facepager.software.informer.com/3.6/), of which 7000 and 6600 data were acquired from Twitter and Facebook, respectively, between September 2019 and 2020.	✓ They analyzed its performance and discovered that the LSVM classifier has the highest precision, recall, and f1-score values of 66%, 66%, and 64%, respectively.
[25]	NLP and PA	✓ They’ve created a news corpus called Afan Oromo. ✓ The general architecture of Afan Oromo fake news detection based on text content is provided. ✓ The article addresses the fundamental obstacles in building text content-based false news detection approaches, as well as potential solutions. ✓ The study compares supervised ML methodologies by taking linguistic features and feature extraction methods into account. ✓ The research sets the door for the development of bogus news identification in Afan Oromo, which would boost user confidence.	✓ Despite the dataset’s shortcomings, the Linear PA with TF-IDF vector and unigram model outperforms the competition with 97.2% of precision, 97.9% of recall, and 97.5% of ROC AUC f1-score.
[35]	DL (including the Bi-GRU and CNN, and attention-based models)	✓ They collected and tagged a dataset of 12,000 news stories to create an automated method for detecting false news.	✓ With an accuracy of 93.92%, a precision of 93%, a recall of 95% (which is smaller than Bi-96 LSTM’s %), and an f1-score of 94%, the CNN model outperforms all other models. ✓ The impact of morphological normalization on Amharic fake news identification was investigated using the top two performing models, and the results demonstrated that normalization harms classification performance, lowering both models’ f1-score from 94–92%.
[29]	DL (including RNN, Bi-LSTM)	✓ They implemented DL models and classified them into pre-defined fine-grained categories to resolve social media fake news for the Afan Oromo language.	✓ On a benchmark dataset, the model can predict with an accuracy of 90%, precision of 90%, recall of 89%, and an f1-score of 89%, outperforming the current state of the art utilizing the Bi-LSTM model.
[19]	MNB classification approach	✓ To best exhibit unambiguous distinctions, the researchers gathered News datasets and accurately categorized them as real and fake news on similar topics.	✓ They used TF, TF-IDF, and TF-IDF of unigram and bi-grams, and discovered that TF of unigram of this model identifies fake news sources with a 96% accuracy, with only minor effects on recall. ✓ For real news accuracy, recall, and f1-score, the confusion matrix was computed at 98.6%, 94%, and 96.2%, respectively, and for fake news precision, recall, and f1-score, at 91%, 97.8%, and 94%, respectively.
[26]	ML classifiers (including, NB, SVM, LR, SGD, RF, and PA Classifier model)	✓ The research has made a substantial contribution to slowing the spread of misinformation in vernacular languages.	✓ The experimental results show a precision of 100% RF for both TF-IDF and Count Vectorizer, a recall of 95% using PA classifier for TF-IDF, and an f1-score of 100% in NB and LR classifier for TF-IDF vectorizer using PA classifier.
[30]	ML (including SVM, NB, and RF) and text mining feature extraction techniques	✓ They gathered posts and comments from Facebook using Face pager’s content retrieval techniques to create the dataset for this investigation.	✓ The experiment produced 21 binary and ternary models for each dataset utilizing two datasets. ✓ Both SVM and NB were outperformed by binary models that used RF with word2vec. ✓ SVM with word2vec, on the other hand, outperforms NB and RF models in classification with a 73% of f1-score, a precision of 76%, and a recall of 75%. ✓ In addition, the ternary SVM model using word2vec produced a 53% of f1-score, which is better than the NB and RF models. ✓ Finally, in both datasets utilized in this study, models based on SVM employing word2vec performed marginally better than NB and RF models.
[31]	RNN (by using LSTM and GRU with word n-grams for feature extraction and word2vec to represent each unique word by vector representation)	✓ Researchers created a tagged massive Amharic dataset by gathering posts and comments from activists who actively participated on Facebook sites.	✓ The RNN-LSTM model produced an improved test of 97.9% for all matrices when used with this dataset and different parameters on GRU and LSTM-based RNN models by feature representation of word2vec.
[36]	Spark ML	✓ Thousands of Amharic posts and comments on suspected social network pages of organizations and individual people’s public pages are crawled as a dataset to execute the various experiments.	✓ The NB approach with the word2Vec feature model outperformed the Facebook social network for Amharic language posts and comments in terms of accuracy, ROC score, and area under precision and recall, with 79.83%, 83.05%, and 85.34% accuracy, ROC score, and area under Precision and Recall, respectively. ✓ For the TF-IDF feature model, the NB achieves better results with 73.02%, 80.53%, and 79.93% for accuracy, ROC score, and area under precision and recall, respectively. ✓ The RF with word2vec feature outperforms the TF-IDF with accuracy, ROC score, and area under precision and recall of 65.34%, 70.97%, and 73.07%, respectively. ✓ TF-IDF is next, with 63.55%, 68.44%, and 69.96%, respectively.
[33]	Classical GBT, RF, DL, RNN-LSTM, RNN-GRU, and word embedding (Word2Vec) model	✓ The suggested method looks into how hate speech detection might be applied to identifying susceptible communities. ✓ Using the example of Amharic text data on Facebook, they were able to identify a potentially vulnerable community in terms of social media hatred. ✓ They gathered and annotated Amharic data to detect hate speech in multicultural Ethiopian society. ✓ Since social media data is very noisy and huge, they used the Apache Spark distributed platform for data pre-processing and feature extraction.	✓ Word2Vec embedding with RNN-GRU had the best performance in the hate speech detection experiments, with an AUC of 97.85%, an accuracy/precision of 92.56%, recall of 97.85%, and an f1-score of 98.42%
[34]	ML (including SVM with TF-IDF, N-gram, and W2vec feature extraction)	✓ Create a tagged hate speech dataset from social media for the Afaan Oromo language. ✓ They create standard Afan Oromo stop word lists, as well as a brief word expansion dictionary. ✓ For Afan Oromo text hate speeches, they create an SVM model. ✓ They put their new model to the test on hate speech identification and came out on top.	✓ Accuracy, f-score, recall, and precision measurements are used to evaluate the experiment. ✓ In all evaluation measures, the framework based on SVM with n-gram combination and TF-IDF achieves 96% (accuracy, f1-score, precision, and recall).

According to the summarization of the relevant related works of the study, Table 1 indicates DL approaches are currently chosen by researchers over ML approaches because of their efficiency in learning from large-scale corpora in unlabeled text.

## Results and discussion

The Internet is one of the most valuable sources of information for its users. Many social media platforms, such as Facebook and Twitter, allow users to be connected. Many types of news and speeches are also spread on these platforms. People now prefer to get their news and speeches from these platforms because they are easy to use and navigate. They also benefited from the ability to post comments, react, and so on these platforms. These advantages entice users to visit these platforms. However, because of their advantages, cyber thieves rely on these platforms as their primary source.

**Fig. 1:**
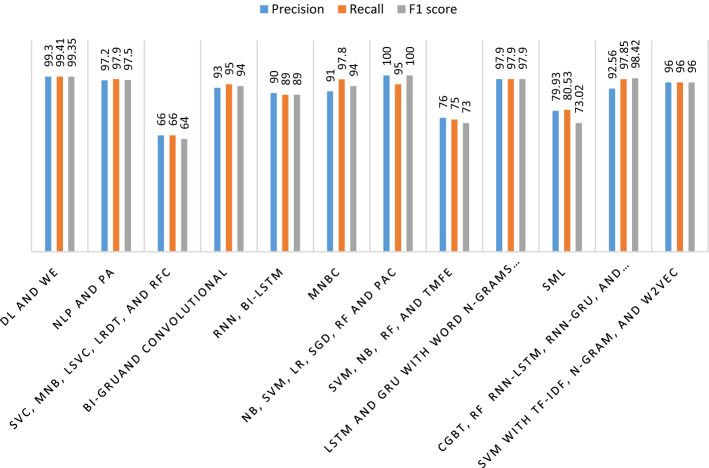
Performance of the implemented approaches

These people can use these platforms to spread fake news and hate speech. There is also the option of sharing posts or news on different networks, which is useful for spreading fake news and hate speech. People started to believe such news and spread it to others. According to [11] and [27], it is impossible to prevent fake news and hate speech from spreading on these social media platforms. Everyone may sign up for these platforms and begin spreading the information. A user can construct a page to act as a news source and propagate fake information. Thus, platforms do not check to see if users are legitimate publishers or not, and they can spread fake information about individuals or organizations in this way. Fake news and hate speech have the potential to hurt society or political parties. As a result, there is a need to detect and combat the spread of fake news and hate speech to save individuals, political parties, or organizations’ reputations [11]. Figure 1 shows an analysis of the assessment metrics used in various studies to assess the performances of all the implemented approaches for hate speech and fake news systems. The most widely used performance measurements are accuracy, precision, recall, and f1-score as expressed in Eqs. (1–4). The model needs higher accuracy, precision, recall, and f1-score to detect fake news and hate speech effectively and efficiently. As a result, these four assessment criteria should be used as performance measures to assess the suggested approaches’ efficiency. Accuracy, recall, f1-score, and precision were the metrics used to evaluate the approach for typical fake news and hate speech detection systems constructed using DL and ML approaches, as shown in Fig. 1 and Table 1.

1 $${\text{Accuracy}} = \frac{{{\text{TP}}}}{{{\text{TR}}}}$$

2 $$\text{P}\text{r}\text{e}\text{c}\text{i}\text{s}\text{i}\text{o}\text{n}=\frac{\text{T}\text{P}}{\text{T}\text{P}+\text{F}\text{P}}$$

3 $$\text{R}\text{e}\text{c}\text{a}\text{l}\text{l}=\frac{\text{T}\text{P}}{\text{T}\text{P}+\text{F}\text{N}}$$

4 $$\text{F}1 \text{s}\text{c}\text{o}\text{r}\text{e}=\frac{2\text{*}\left(\text{P}\text{r}\text{e}\text{c}\text{i}\text{s}\text{i}\text{o}\text{n}\text{*}\text{R}\text{e}\text{c}\text{a}\text{l}\text{l}\right)}{\text{P}\text{r}\text{e}\text{c}\text{i}\text{s}\text{i}\text{o}\text{n}+\text{R}\text{e}\text{c}\text{a}\text{l}\text{l}}$$

where TP represents the number of correctly categorized fake news in the real news category, FP represents the number of incorrectly classified fake news in the negative news category, FN represents the number of fake news incorrectly classified in the negative news category, and TR represents the total number of the languages news and speech in the test data. In Fig. 1, the evaluation metrics are different even from the same DL and ML approaches. Accordingly, this review work presents the approaches from which the best evaluation performances have been achieved.

**Fig. 2:**
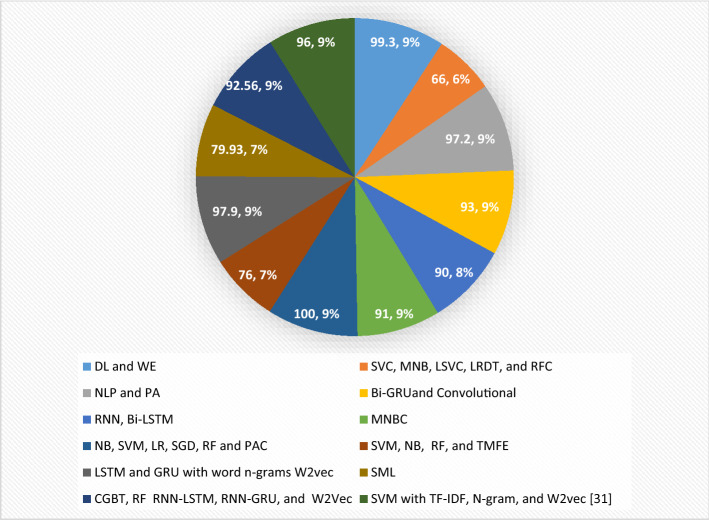
Distribution of implemented approaches

Based on the reviewed research work, the researcher discovered that most researchers achieved better evaluation results in DL approaches than in ML approaches for developing fake news and hate speech detection systems, as shown in Fig. 2. It can be observed that SVM with TF-IDF, N-gram, and W2vec is 9%. DL and WE account for 9% of the total, while SVC, MNB, LSVC, LRDT, and RFC account for 6%. NLP and PA are 9%, while Bi-GRU and Convolutional are 9%. RNN, Bi-LSTM is 8%, MNBC is 9%, NB, SVM, LR, SGD, RF, and PAC is 9%, SVM, NB, RF, and TMFE is 7%, LSTM, and GRU with word n-grams W2vec is 9%, SML is 7%. CGBT, RF, RNN-LSTM, RNN-GRU, and W2Vec are 9%. In Fig. 2, most researchers have combined different approaches, such as DL and ML, to detect and combat fake news and hate speech on various social media platforms. According to the work reviewed in this study, using ML approaches produced the best results due to their efficiency in learning from large-scale unlabeled text corpora.

**Fig. 3:**
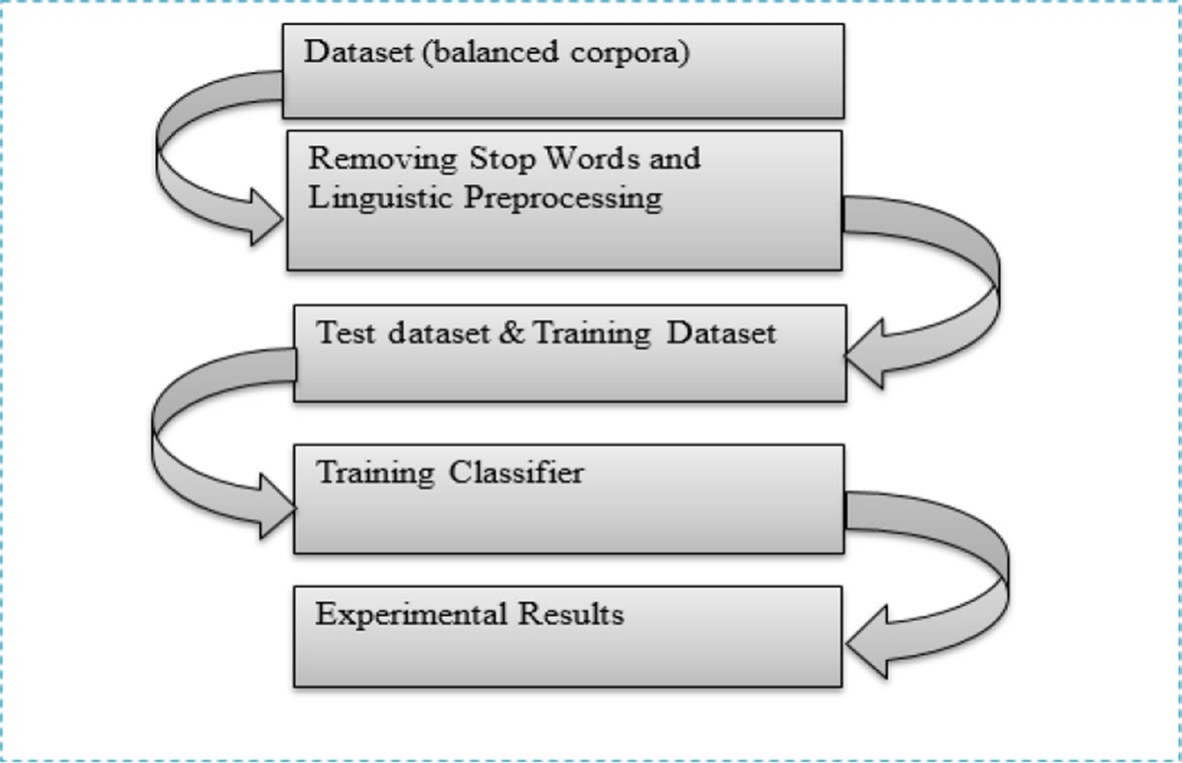
Dataset training process for fake news and hate speech systems

According to the observed results, future researchers in the area can use ML approaches with great consideration of large-scale datasets to increase the performance evaluation metrics (detection rates) of the systems. According to researchers in [11, 27, 32] the various DL and ML approach trained to recognize fake news and hate speech should follow the process depicted in Fig. 3.

There has been no research work on detecting and combating fake news and hate speech that has produced 100% accuracy for Ethiopian languages. As a result, all researchers in the field have used various fake news and hate speech detection approaches, which have proven effective in detecting fake news and hate speech on various social media platforms. Accordingly, the performance of any fake news and hate speech detection system for the Ethiopian languages depends on a detailed examination of the datasets, including the size of the data and the platform from which the data has been collected. The fake news and hate speech detection system, which incorporates all essential features from various social media platforms, can be used to build the best and most appropriate fake news and hate speech detection model capable of detecting all types of features for Ethiopian languages. Finally, testing fake news and hate speech detection systems on large-scale text collections derived from various sources that can represent all features better than a small-scale pattern will improve the accuracy of the fake news and hate speech detection systems for Ethiopian languages.

## Conclusion and recommendation

This review provides new researchers with up-to-date knowledge, recent researchers’ inclinations, and advancements in the arena by providing a comprehensive assessment of fake news and hates speech detection approaches based on DL and ML approaches. A systematic review strategy was used as a method to prioritize and select Ethiopian language research works in the field of AI-based fakenews and hate speech detection systems. Based on the analyzed research work, the theoretical concepts of fake news and hate speech detection approaches have been thoroughly presented.

The approaches used by each research work have then been presented, as well as all the research’s evaluation metrics regarding the detection models’ competence and difficulty. According to this review work, new research reveals that using a DL approach improves the system’s performance regarding all detection rates. Research work that has been implemented using DL approaches with great consideration of the collected datasets will produce the best performance in all evaluation metrics (detection rates) of the system. Finally, based on the comparative results of the implemented approaches, DL approaches have been recommended for other Ethiopian languages to increase the performance of all evaluation metrics from different social media platforms. Additionally, as the review results indicate, the combination of DL and ML approaches with a balanced dataset can improve the detecting and combating performance of the system.

## Data Availability

Not applicable.
